# A comparative study of mirror self-recognition in three corvid species

**DOI:** 10.1007/s10071-022-01696-4

**Published:** 2022-09-29

**Authors:** Lisa-Claire Vanhooland, Anita Szabó, Thomas Bugnyar, Jorg J. M. Massen

**Affiliations:** 1 Department of Behavioural and Cognitive Biology, University of Vienna, Djerassiplatz 1, 1030 Vienna, Austria; 2 Department of Zoology, Charles University, Prague, Czech Republic; 3 Animal Behaviour and Cognition, Department of Biology, Utrecht University, Utrecht, The Netherlands

**Keywords:** Mirror response, Self-awareness, Mirror-mark test, Azure-winged magpies, Common raven, Carrion crow

## Abstract

Mirror self-recognition (MSR) assessed by the Mark Test has been the staple test for the study of animal self-awareness. When tested in this paradigm, corvid species return discrepant results, with only the Eurasian magpies and the Indian house crow successfully passing the test so far, whereas multiple other corvid species fail. The lack of replicability of these positive results and the large divergence in applied methodologies calls into question whether the observed differences are in fact phylogenetic or methodological, and, if so, which factors facilitate the expression of MSR in some corvids. In this study, we (1) present new results on the self-recognition abilities of common ravens, (2) replicate results of azure-winged magpies, and (3) compare the mirror responses and performances in the mark test of these two corvid species with a third corvid species: carrion crows, previously tested following the same experimental procedure. Our results show interspecies differences in the approach of and the response to the mirror during the mirror exposure phase of the experiment as well as in the subsequent mark test. However, the performances of these species in the Mark Test do not provide any evidence for their ability of self-recognition. Our results add to the ongoing discussion about the convergent evolution of MSR and we advocate for consistent methodologies and procedures in comparing this ability across species to advance this discussion.

**Supplementary Information:**

The online version contains supplementary material available at 10.1007/s10071-022-01696-4.

## Introduction

Self-recognition is considered one of the milestones of cognitive development and has been argued to play a crucial role in the development of self-awareness in human and non-human animals (Rochat et al. 2012). Self-recognition in non-human animals has most commonly been studied by investigating an individual’s ability to recognize itself in a mirror. Mirror self-recognition (MSR) can conclusively be attributed to an individual when they pass the mark test (Gallup 1970). In this test, a mark is inconspicuously placed on an out-of-view body part. Attempts to touch, inspect, or remove the mark by utilizing the mirror indicate the individual’s capacity to make the association between its mirror reflection and itself. According to the social cognition hypothesis (Gallup 1982; Krachun et al. 2019), MSR reflects the individual’s awareness of its own behaviors and mental states, which would constitute the basic building block for higher cognitive abilities such as Theory of Mind (ToM) or empathy (Gallup 1982, 1985).

The mark test has been broadly used to study the phylogenetic distribution of mirror self-recognition and self-awareness in mammals (i.e., elephants (Plotnik et al. 2006), horses (Baragli et al. 2017), pandas (Ma et al. 2015), marine mammals (Delfour and Marten 2001; Reiss and Marino 2001), primates (Paukner et al. 2004; Roma et al. 2007; Suddendorf and Collier-Baker 2009; Chang et al. 2015)), fish (cichlids: Hotta et al. 2017, mantas: Ari and D’Agostino 2016, cleaner wrasses: Kohda et al. 2019, 2022), birds (see Brecht et al. (2020) for review), and invertebrates (squids: Ikeda and Matsumoto 2007, ants: Cammaerts and Cammaerts 2015). Despite being debated (Anderson and Gallup 2015; Gallup and Anderson 2018, 2020), these studies show that this ability evolved independently in great apes (humans (Amsterdam 1972), chimpanzees (Gallup 1970; Povinelli et al. 1997), bonobos (Westergaard and Hyatt 1994; Walraven et al. 1995), orangutans (Lethmate and Dücker 1973; Suarez and Gallup 1981; Miles 1994), gorillas (Patterson and Cohn 1981; Parker et al. 1994; Posada and Colell 2007)), dolphins (Reiss and Marino 2001; Morrison and Reiss 2018), elephants (Plotnik et al. 2006), cleaner wrasses (Kohda et al. 2019, 2022), two corvid species (i.e., Eurasian magpies (Prior et al. 2008), and Indian house crows (Buniyaadi et al. 2020)).

These findings have advanced the idea that self-recognition might have evolved as a by-product in big-brained, highly social, and cognitively developed animals. Indeed, the body of comparative research seems to largely support the social cognition hypothesis as most species that pass the Mark test also show evidence of more complex cognitive abilities. This theory was recently further substantiated by a study in chimpanzees in which the individuals that exhibited signs of mirror self-recognition overall also performed better in socio-cognitive tasks (Krachun et al. 2019). Yet, recent findings that claim that ants (Cammaerts and Cammaerts 2015) and cleaner wrasses (Kohda et al. 2019) can pass the Mark Test challenge these assumptions and led to a more gradualist conception of animal self-awareness (de Waal 2019).

While the mark test only allows for two outcomes (pass or fail), the observation of the types of behaviors exhibited towards the mirror can provide more information about the individual’s understanding of the mirror and a more gradualist approach to self-awareness (de Waal 2019). When first confronted with mirrors, many animal species display social (agonistic or affiliative) behaviors towards their reflection. With more experience, some will start exhibiting explorative and contingent behaviors towards the mirror and only a few species will further start exhibiting self-directed behaviors (i.e., exploring body parts out of sight for them when the mirror is not present), a behavior often correlated with the successful passing of the mark test (Povinelli et al. 1993). This sequence of behaviors observed in non-human animals parallels the stages undergone by human infants in their development (Rochat 2003). Rochat (2003) identifies five levels of self-awareness in the gradual development of mirror understanding of infants ranging from a state of mirror confusion (i.e., lack of self-awareness) to a state of self-consciousness (i.e., an awareness of self as perceived by others). In this categorization, the state of confusion between the mirror and the environment is notably expressed by social responses towards the mirror as well as attempts to pass through the mirror, a type of behavior also frequently noted during initial mirror encounters amongst animals (e.g., Pickering and Duverge 1992; Kusayama et al. 2000), and which some species will persist exhibiting irrespective of their experience with mirrors. On the first level of self-awareness, the individual understands the difference between the reflection and the environment and observes the contingency between its own movements and the reflection which on the second level is followed by an understanding of the connection between the proprioceptive experience of the movement and the reflected image (as seen in contingency checking behaviors, which have also been observed in non-human species Povinelli et al. 1993; Ari and D’Agostino 2016; Vanhooland et al. 2020)). An alternative approach to investigate these levels of mirror understanding seen in the non-human animal literature has been to look at a species’ ability to use a mirror to locate, e.g., food (Anderson 1986; Pepperberg et al. 1995; Broom et al. 2009; Medina et al. 2011) or conspecifics (Itakura 1987). On the third level, individuals are able to identify themselves in the reflection and show signs in line with self-recognition (i.e., self-directed behaviors and mark removals in the mark test), as observed in very few non-human animal species passing this task (as discussed above). Finally on level four and five, individuals gain, respectively, the permanence of the self across time and space (e.g., being able to recognize a younger self in a photograph), and the self-consciousness of understanding that they are also perceived by the individuals around them giving rise to, e.g., self-conscious emotions (Rochat 2003), which have received little attention in non-human animals studies.

Reputed for their big brains (Güntürkün and Bugnyar 2016; Olkowicz et al. 2016), complex social lives (Bugnyar 2013; Massen et al. 2014), and cognitive capacities rivalling those of apes (Emery 2004; Emery and Clayton 2004), corvids represent an interesting case of cognitive convergence (Seed et al. 2009; Güntürkün and Bugnyar 2016; Baciadonna et al. 2021). Specifically, several studies showing metacognitive (Goto and Watanabe 2012; Watanabe and Clayton 2016; Watanabe 2018), theory of mind-like (Bugnyar and Kotrschal 2004; Dally et al. 2010; Bugnyar 2011), or mental time travel (Clayton and Dickinson 1998; Raby et al. 2007; Kabadayi and Osvath 2017) abilities in some corvids would prompt us to also suspect higher levels of self-awareness in this taxonomic group making them good models for the study of self-concepts. Yet, when exploring the MSR abilities of corvids, only very few species seem to be able to pass the mark test.

In fact, two corvid species (i.e., the Eurasian magpie and the Indian house crow) are the only avian species to have, to date, passed the mark test (Prior et al. 2008; Buniyaadi et al. 2020). However, attempts to replicate these findings in the Eurasian magpies (Soler et al. 2020) or the Indian house crow (Parishar et al. 2021) as well as studies on other corvid species such as jackdaws (Soler et al. 2014), Clark’s nutcrackers (Clary and Kelly 2016), California scrub jays (Clary et al. 2020), azure-winged magpies (Wang et al. 2020), large-billed crows (Kusayama et al. 2000), New Caledonian crows (Medina et al. 2011), carrion crows (Brecht et al. 2020; Vanhooland et al. 2020), and hooded crows (Smirnova et al. 2020) failed to render any conclusive evidence on these species’ abilities of MSR. Thus, indicating that the pre-requisites defined to date (i.e., a high encephalization index, high social complexity, and advanced cognitive abilities) are not sufficient to predict MSR. Yet, the origin of these divergent results has barely been addressed and an explanation for the interspecies differences of phylogenetically closely related species is lacking in corvids. It, therefore, remains unclear which factors, be they methodological, cognitive or ecological, drive positive results in MSR in corvids, making more comparative studies an imperative to better understand the mechanisms underlying this cognitive ability.

On the one hand, little is known about the evolutionary drivers underlying the emergence of mirror self-recognition in corvids and whether this ability is the result of a divergent or convergent evolution in different branches of the Corvidae family. Nor do we precisely know which ecological factors or other cognitive make-up would underlie such a convergent evolution in corvids. For instance, in children, MSR has been found to emerge during the second year of life in synchrony with the ability to express prosociality and the ability to imitate (Bischof-köhler 2012). Although there are only few comparative studies of such higher cognitive abilities in corvids, some studies show interspecies differences in the cognitive and emotional abilities known to co-emerge with MSR in infants, e.g., in prosocial tendencies (Horn et al. 2020).

On the other hand, methodological differences in procedures (e.g., type of marking, marking procedure, and amount of pre-experience with mirrors) or test subjects (age, proprioceptive development, rearing, and housing) complicate interspecies comparisons of mirror responses and the performances in the mark test, as we do not yet know how these factors affect the birds’ responses (an overview of the methodological differences between mirror self-recognition studies in corvids is provided in Table 1). Particularly, the issue of testing singly housed and potentially socially deprived animals in a procedure that possesses an inherently social component, as well as the testing of wild-caught animals in very small enclosures after human handling, must be taken into account when regarding the measurement of potentially non-typical behavioral responses (e.g., due to augmented stress levels).

**Table 1:** Methodological review of the to-date tested corvid species including specifications on housing, rearing, age, and performance in the test

Species	References	Number of IDs in study (*n*)	Duration of mirror exposure (excluding the MT) [in min]	Number of IDs Passing MT	Age	Housing	Rearing	Mark type (including size and weight)	Mark colour	Marking method	Mark location	Visible mark control	Size of Experimental chamber^a^ (length × width × height)	Size of mirror (in cm)
Jackdaw (*Corvus monedula*)	Soler et al. (2014)	9	270	0/9	Adult	Communal	6 hand-raised, 3 wild-caught	Stickers: ∅ 6 mm, ~ 2.4 mg	Yellow, red	Restrained bird	Throat	No	160 × 100 × 80 cm	50 × 60
Carrion crow (*Corvus corone *ssp.)	Vanhooland et al. (2020)	8	390–710	0/7	Adults	Communal	Hand-raised	Liquid food colouring	Red, blue	Voluntary participation after training	Throat	Yes	3.7 × 7 × 5m	50 × 50
Carrion crow (*Corvus corone corone*)	Brecht et al. (2020)	12	3840 (~ 64 h)	0/12	8 adults 2 juveniles 2 sub-adults	Communal	Hand-raised	Liquid chalk	Yellow	Restrained bird	Throat	Yes	105 × 105 × 140 cm	80 × 80
Hooded crow (*Corvus cornix*)	Smirnova et al. (2020)	6	240	0/6	> 2 years	Communal	n.a	Square pieces of newsprint: 5 × 5 mm, 4 mg	Red	Restrained bird	Head	No	120 × 45 × 50 cm	35 × 45
New Caledonian crow (*Corvus moneduloides*)	Medina et al. (2011)	10	30	–	2 adults 8 juveniles	Communal	Wild-caught	–	–	–	–	–	4 × 5 × 3 m	40 × 50
Indian house crow (*Corvus splendens*)	Buniyaadi et al. (2020)	6	120	4/6	Adult	Single	Wild-caught	Sticker: ∅ 7 mm, ~ 4.5 mg	Red, yellow	Restrained bird	Throat	No	100 × 80 × 80 cm	45 × 30
Indian house crow (*Corvus splendens*)	Parishar et al. (2021)	5	240	0/5	Adult	Single	Wild-caught	Sticker: ∅ 9 mm, 12-13 mg	Yellow	Restrained bird	Head and throat	Yes	76 × 53 × 86 cm	30 × 20
Common raven (*Corvus corax*)	Vanhooland et al., in rev^b^	10	240–260	0/10	9 adults 1 juvenile	Communal	9 hand-raised, 1 captive-bred parent-raised	Liquid food colouring	Red	Voluntary participation after training	Head and throat	Yes	8 × 7 × 5 m	50 × 50
Jungle crow (*Corvus macrorhynchos*)	Kusayama et al. (2000)	4	180	–	2 juveniles 2 sub-adults	Single	Wild-caught	–	–	–	–	–	90 × 90 × 60 cm	﻿45.7 × 35.7
Azure-winged magpie (*Cyanopica cyanus*)	Wang et al. (2020)	7	150–300	0/7	Juvenile	Single	Hand-raised	Vegetable powder	Red	Restrained bird	Throat	Yes	60 × 40 × 40 cm	28 × 28
Azure-winged magpies (*Cyanopica cyanus*)	Vanhooland et al., in rev^b^	6	320–720	0/6	Adult	Communal	4 hand-raised, 2 captive-bred parent-raised	Liquid red food colouring	Red, yellow	Voluntary participation after training	Throat	Yes	2.25 × 3 × 3 m	30 × 30
Eurasian magpie (*Pica pica*)	Prior et al. (2008)	5	250	2/5	Adult	n.a	Hand-raised	Stickers: ∅8 mm, 16 μg	Yellow, red, blue	Restrained bird	Throat	No	Mirror preference test: 120 × 120 × 100 cm Mark test: 120 × 60 × 100 cm	55 × 40
Eurasian magpie (*Pica pica*)	Soler et al. (2020)	8	270	0/8	Adult	Communal	Wild-caught	Stickers: ∅ 6 mm, 2.4 mg; ∅ 10 mm, 3.96 mg	Yellow, red	Restrained bird	Throat	No	160 × 100x80 cm	60 × 50
Clark’s nutcracker (*Nucifraga columbiana*)	Clary and Kelly (2016)	17	2160–2220 (37 h)	1/10	Adult	Single	n.a	Stickers ∅ 6 mm, ~ 6.3 mg	Red	Restrained bird	Throat	No	62 × 31.75 × 66.5 cm	65 × 62 (37 × 62)
California scrub jay (*Aphelocoma californica*)	Clary et al. (2020)	7	380	0/7	Adult	Single	Wild-caught	Stickers ∅ 6 mm, ~ 6.3 mg	Red	Restrained bird	Throat	No	62 × 31.75 × 66.5 cm	65 × 62

Not all studies on mirror responses in corvids also included a mark test (MT) in which case the corresponding cells were marked with a dash (“–”). “n.a.” indicated cases for which the required information was not available in the publication

^a^i.e., space the individual had to roam around in when presented with a mirror in its environment

^b^data from the present study

In this study, we provide the first comparative study on mirror responses and mirror self-recognition of three corvids species which were part of captive colonies and had similar keeping (i.e., in social pairs or groups) and rearing backgrounds. We will present in this paper original data for two corvid species: common ravens (*Corvus corax*) and azure-winged magpies (*Cyanopica cyanus*), and will compare the obtained results to the mirror responses of carrion crows (*Corvus corone *ssp.) previously tested (Vanhooland et al. 2020) following similar procedures. All three species are part of the corvid family and therefore share the characteristics attributed to this taxonomic group. Thus, all possess the established pre-requirements for MSR (e.g., object permanence), but also possess unique traits making them interesting models to investigate possible evolutionary drivers of mirror self-recognition.

Common ravens form selective close long-term social bonds as non-breeders (Boeckle and Bugnyar 2012) and later become territorial monogamous breeders (Boucherie et al. 2019). Non-breeders form ‘open’ groups with moderate-to-high degrees of fission–fusion dynamics (Bugnyar 2013; Loretto et al. 2017; Boucherie et al. 2019) and display close coordination during foraging (Hendricks and Schlang 1998), conflict resolution (Fraser and Bugnyar 2010, 2011), as well as in experimental setups like in the loose string paradigm (Massen et al. 2015, 2020b), yet display low levels of prosocial behaviors (Di Lascio et al. 2013; Massen et al. 2015; Lambert et al. 2017; Horn et al. 2020). They have further been shown to possess abilities of future planning (Kabadayi and Osvath 2017), perspective taking (Bugnyar et al. 2004; Bugnyar 2011, 2013), and tactical deception (Bugnyar and Kotrschal 2002, 2004), thus demonstrating aspects of Theory of Mind. In contrast, the azure-winged magpie nests in colonies and is a cooperatively breeding species (Komeda et al. 1987; Cockburn 2006) and has been shown to possess strong prosocial tendencies (Horn et al. 2016; Massen et al. 2020a) commonly related to high social tolerance (Horn et al. 2016). Finally, the carrion crow shares attributes with both of the above-mentioned species. Carrion crows are phylogenetically very closely related to the common raven with which they share an ecological niche. Carrion crows and common ravens have similar social structures and life histories. Although they are most commonly territorial breeder like the common ravens, carrion crows have been shown to become cooperative breeders under certain environmental conditions (Baglione et al. 2002a, b, 2016; Marcos et al. 2006) and to be moderately prosocial (Horn et al. 2016, 2020, 2021).

We examined 10 common ravens and 6 azure-winged magpies in a classical two-phased mirror self-recognition paradigm, closely following the procedure of Vanhooland et al. (2020), consisting first of a phase of mirror exposure familiarizing the birds with the mirror and testing for mirror preference, followed by a mark test. In the first phase, the azure-winged magpies and ravens were exposed to three conditions: a mirror, a non-reflective silver foil, and a wooden board. In the mark test, all subjects were tested in four conditions (mirror-mark, mirror-sham, wood-mark, and wood-sham). The ravens and azure-winged magpies’ results were subsequently compared to the performances of the carrion crows (Vanhooland et al. 2020). We expected both species to prefer spending time at the mirror, as this is a trend commonly found in birds, and to display all behavioral categories more in the mirror condition than in the other two conditions. Due to their cooperative breeding lifestyle, prosocial tendencies, and consequently supposedly increased social tolerance, we expected less agonistic social interaction with the mirror reflection from the azure-winged magpies than from the ravens, which we would expect to display stronger reactions to the “unfamiliar conspecific” in the mirror due to the territoriality of breeders and their strict hierarchies in non-breeder groups (Boucherie et al. 2022). We further expected the azure-winged magpies to be more explorative than the ravens as a consequence of lower levels of neophobia (Miller et al. 2022). In addition, if the birds understood that their reflection is not a conspecific, we would expect them to display contingency checking behaviors (as a precursor of MSR) and self-directed behaviors (indicative of MSR) during the mirror exposure phase. Finally, if ravens and/or azure-winged magpies are capable of mirror self-recognition, due to their generally high intelligence and good performance on ToM-like tasks, or due to their prosocial tendencies, respectively, we expected the birds to perform mark-directed behaviors only in the mirror-mark condition of the mark test.

## Methods

### Subjects

In this study, we tested ten common ravens (*Corvus corax;* 4 M, 6F) and six azure-winged magpies (AWM, *Cyanopica cyanus*, 2 M, 4F). All subjects that participated in this study (except for one juvenile female raven) were adult birds that were born and raised in captivity and very habituated to human interaction (Table 2 for specifications). The common ravens were housed at the Haidlhof research station (Bad Vöslau, Austria) and kept in pairs (*n* = 5) or groups (*n* = 5) depending on their breeding status, i.e., breeders were kept as pairs in separate aviaries (dimensions: 10mx8mx5m); non-breeders were group-living in a common aviary (dimensions: 18 m x 15 m x 5 m). The azure-winged magpies were all kept as a group in a single aviary (dimensions: 4.25 m x 3 m x 3 m) at the Animal Care Facility of the Department of Behavioral and Cognitive Biology at the University of Vienna. All birds had ad libitum access to food and water over the entire course of this study.

**Table 2:** Specifications of the test subjects of this study (ID, sex, year of birth, housing, and breeding status) and on their test condition in the Mark test (i.e., the location of the coloured mark on the body and the colour of the applied mark)

Species	ID	Sex	YoB	Housing	Breeding status	Mark position	Mark colour
Raven	Astrid	F	2010	Pair	Breeder	Head	Red
Raven	Horst	M	2012	Pair	Breeder	Throat	Red
Raven	Joey	F	2010	Pair	Breeder	Head	Red
Raven	Rocky	M	2012	Pair	Breeder	Head	Red
Raven	George	M	2012	Group	Non-breeder	Throat	Red
Raven	Nobel	F	2012	Group	Non-breeder	Throat	Red
Raven	Munia	F	2014	Group	Non-breeder	Throat	Red
Raven	Aramis	F	2014	Group	Non-breeder	Throat	Red
Raven	Ikarus^a,b^	F	2017	Group	Non-breeder	Head	Red
Raven	Laggie	M	2012	Pair	Breeder	Throat	Red
AWM	Anakinb	M	2015	Group	Non-breeder	Throat	Yellow
AWM	BB8	F	2016	Group	Non-breeder	Throat	Red
AWM	Chewieb	F	2015	Group	Non-breeder	Throat	Red
AWM	Kylo	M	2016	Group	Non-breeder	Throat	Yellow
AWM	Rey	F	2016	Group	Non-breeder	Throat	Red
AWM	Poe	F	2016	Group	Non-breeder	Throat	Red

^a^Was still a juvenile when the study took place

^b^Parent-raised individuals, all others were hand-raised by experienced researchers

### Ethical approval

This study was conducted in compliance with the Austrian Animal Experimentation Act as well as the ASAB ethical guidelines and was approved by the ethical board of the University of Vienna (2022–005). The conducted experiment was non-invasive in nature and the birds’ participation voluntary (i.e., entering the experimental compartment, getting marked, etc.). In case a bird started showing any signs of distress, the test sessions were terminated immediately.

### Materials and methods

The procedure followed in this study derives from the procedure previously implemented in carrion crows by Vanhooland et al. (2020), with some minor deviations detailed below.

### Apparatus

**Fig. 1:**
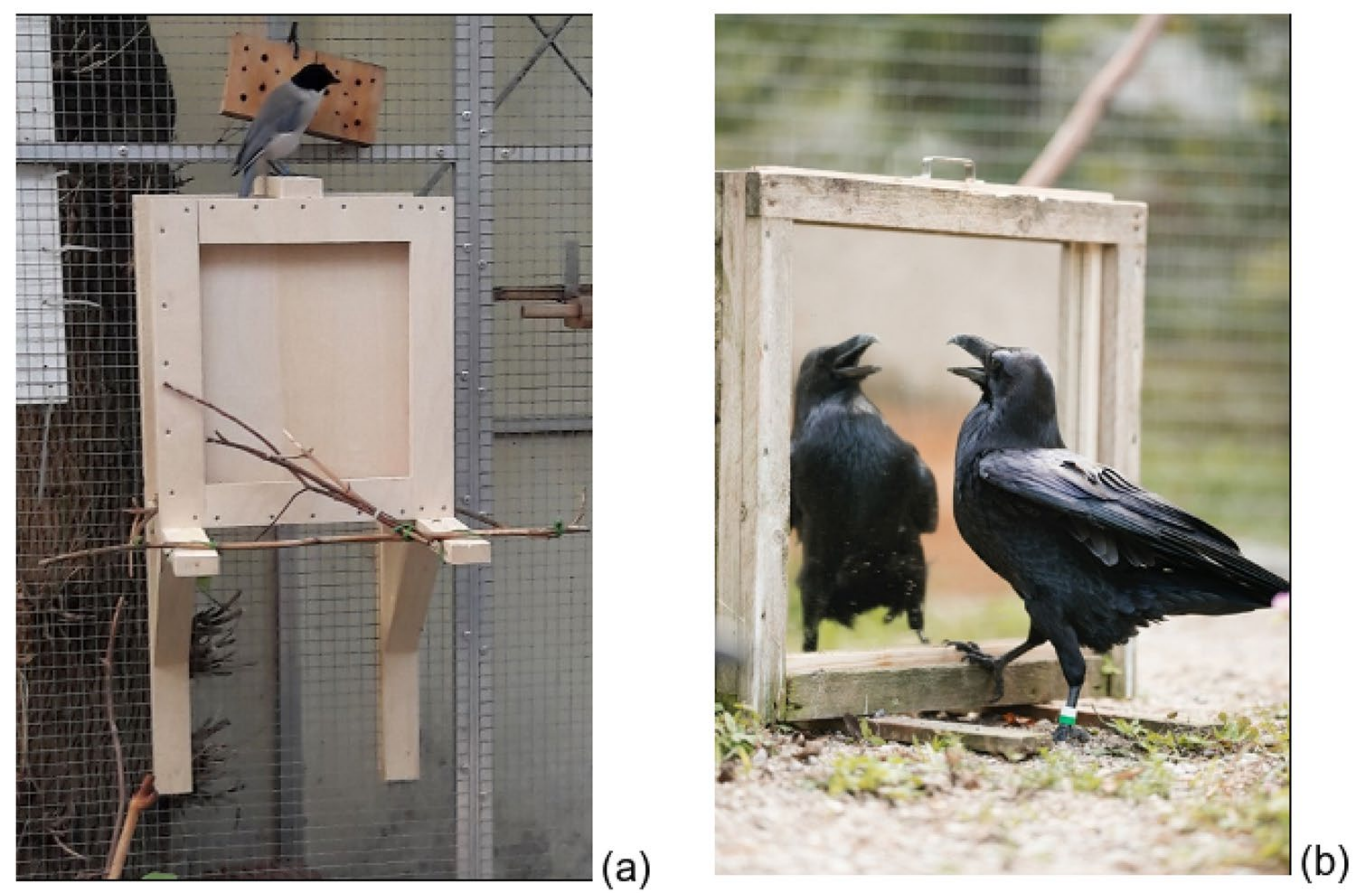
Apparatus of the azure-winged magpies (**a**) and the common ravens (**b**) (© I. Grubert)

The apparatus consisted of a wooden frame in which a mirror, a wooden board, or a board covered with a shiny plastic silver foil could be inserted. While the wooden board did not possess any reflective properties and was a material familiar to the birds, the mirror and the silver foil constituted new materials with shiny properties, of which only the mirror provided a perfect reflection of the bird’s body. The size of the apparatus was adapted to the species body size and was such that the birds could see their entire body in the mirror, i.e., each mirror was about two times the body length of a bird: 50 cm × 50 cm for the common ravens, and 30 cm × 30 cm for the azure-winged magpies. The size of the two control boards (wooden board and silver foil) matched the size of the mirror used in the experiment. The apparatus was set up in a home-range compartment of the birds and stayed there over the entire length of the experiment, so that the empty apparatus could be approached by the birds at any time. The positioning of the apparatus within the aviary was adapted to the species’ preferred way of access (i.e., whether they generally feel more comfortable approaching an object while perched or on the ground). Therefore, the apparatus was installed on the ground for the common ravens and fixed to the side of the aviary 1.5 m off the ground for the azure-winged magpies and furnished with sufficient perching opportunities to allow the birds to walk around the apparatus and inspect all sides of the apparatus (Fig. 1).

### Procedure

All test sessions were conducted in one of the birds’ home-range compartments (compartment dimensions for the ravens: 8 × 7 × 5m; for the azure-winged magpies: 2.25 m x 3 m x 3 m). All birds were habituated to the apparatus prior to the start of the experiment, yet slightly deviating from Vanhooland et al. ’s (2020) procedures, the ravens and AWM were habituated to an empty apparatus (contrarily to the apparatus containing a silver foil during the habituation period in Vanhooland et al. 2020), as the silver foil was used as an additional test condition in the present study, to account for the effect of novelty and shininess of the object in the frame.

Before conducting the mirror-mark test, all birds gained experience with the mirror and the two control boards (wood and silver foil), first through group exposures to the apparatus followed by individual sessions.

A group exposure session was defined as a session in which the entire social unit of the animals had ad libitum access to the apparatus during the entire session. Social units consisted either of a pair (individual with pair-bonded mate), a family unit (pair with this year’s offspring), or of the non-breeder group the animal belonged to. In individual exposure sessions, the focal individual was separated from its social unit for the length of the session and had ad libitum access to the apparatus. Group exposure sessions mainly aimed at facilitating the habituation to the apparatus and to overcome the initial neophobia. All non-breeding birds started receiving individual sessions when at least all individuals but one from the group approached the apparatus in all 3 conditions (mirror, silver foil, and wood) during group exposure (this resulted in the non-breeding ravens receiving one group session of 20 min in each condition, while the azure-winged magpies received 2 sessions in each condition for a total of 120 min). Contrarily, breeding birds’ family units were not split up as long as the chicks were still with their parents, as a separation between the juveniles and parents would cause severe stress to the birds.

All individuals first received 4 sets of sessions, each set consisting of 2 mirror, 1 wood, and 1 silver foil session to reproduce the type of exposure given to the crows by Vanhooland et al. (2020) and accelerate the exposure process to the unknown mirror, followed by 4 mirror, 4 wood, and 4 silver foil sessions before the mark test. The order in which these three conditions were presented to the animals within a set was randomized; resulting in each bird receiving a total of at least 12 mirror, 8 wood, and 8 silver foil 20-min sessions and receiving supplemental sessions if they had not spent at least 10 min in front of the mirror, but no more than a total of 30 mirror sessions. As we did not separate birds from their family unit during breeding, raven breeders were mostly exposed to the apparatus in their family units rather than individually (i.e., for the initial 4 sets) and started individual exposure sessions only once the chicks had left their parents (i.e., for the remaining 4 exposure sessions in each condition prior to the mark test).

Each test session started after an experimenter placed treats in front of the apparatus and ended after the 20 min with the bird’s return to the group. The apparatus was baited at every session to control for the individual’s willingness to approach the apparatus, allowing the distinction between a lack of interest towards the apparatus and a neophobic response (as a lack of interest would result in the collection of the baits but no further time spend at the apparatus, while a neophobic response would result in no collection of the baits). Consequently, sessions in which the birds did not approach the apparatus were repeated on a following day.

## Mirror-mark test

### Marking procedure

**Fig. 2:**
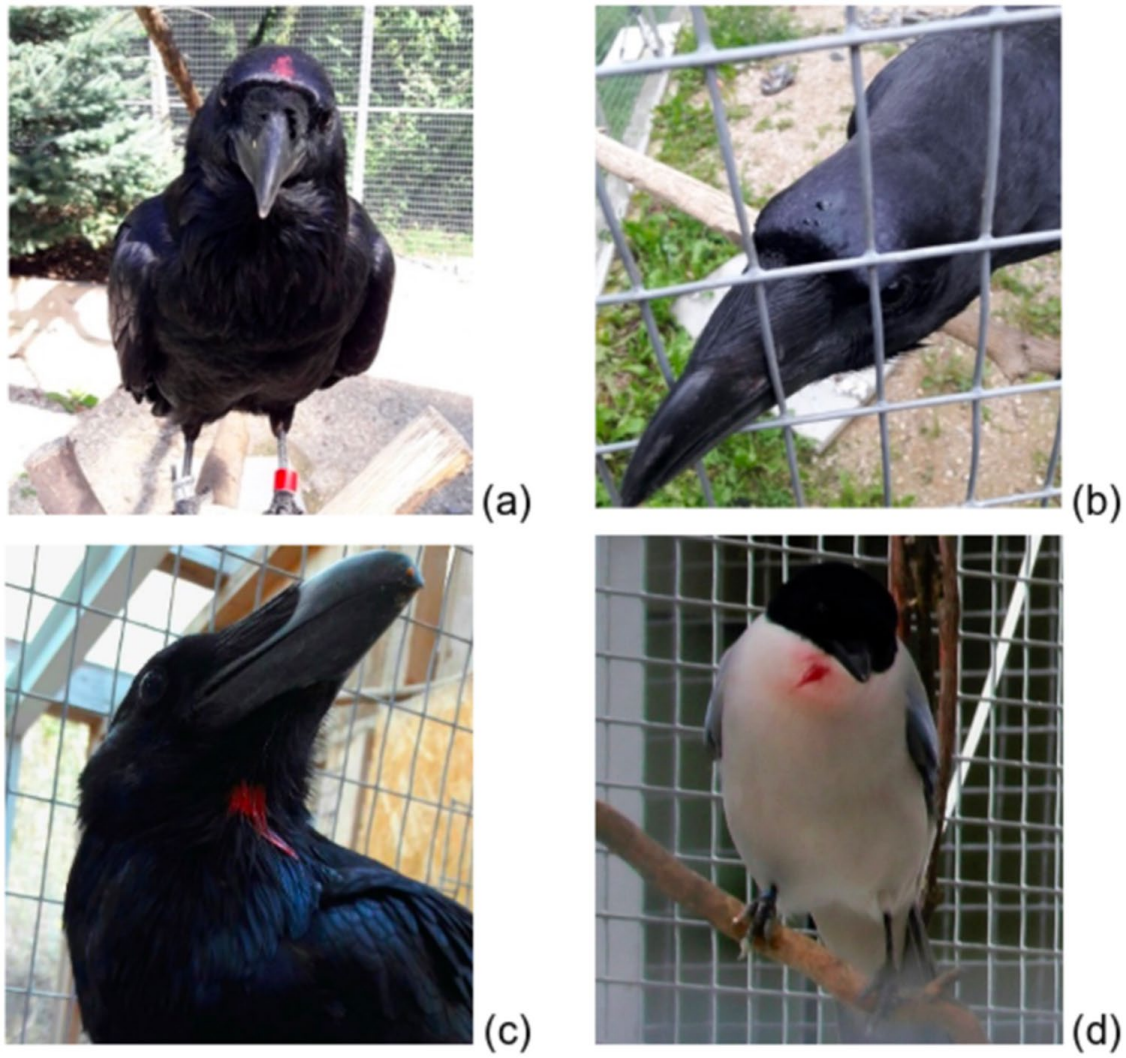
Markings of the ravens and azure-winged magpies: coloured markings on the raven’s and azure-winged magpie’s head and throat (**a**, **c**, **d**) and sham marking (**b**)

Prior to the mirror-mark test, all birds were trained to participate voluntarily in the marking procedure. Birds were trained to approach the experimenter holding a brush and allow them to touch the top of their heads or throats (for the mark test) as well as their belly or wings (for the visible mark control) with this brush. The placement of the mark was adapted to the bird’s preferences during training, to ensure continued cooperation from the bird. The brush hairs had been dyed to match the colour of the dye used in the mark test as to not cue the birds on the test condition, although this dye was already dry, and thus, during the habituation phase, would not leave a mark on the birds. Actual markings, i.e., during the tests, were applied using a mix of glycerine and food colouring (for the coloured marks) and pure glycerine (for the sham marks) (Fig. 2), a method previously successfully implemented by Vanhooland et al. (2020) and shown to seemingly not provide somatosensory cues on the mark’s location to the bird. The ingredients used to make the marks were safe for consumption and water soluble.

### Test

The Mark test consisted of four conditions in which the apparatus would either contain the mirror or the wooden board and the birds were marked with either a coloured or a sham mark on a body part not visible to the bird (see marking procedure). The azure-winged magpies were further tested in the two marking conditions while the apparatus contained the silver foil as they, contrarily to the common ravens, seemed more interested in the silver foil than the wooden control board during the exposure sessions. Each bird was tested twice in each condition and each test session lasted 20 min. The order of the test sessions was randomized within subjects.

### Visible mark control

We further implemented visible mark control conditions in which the marks were applied to a body part the birds could see without the assistance of a mirror, to test their susceptibility to remove such marks from their bodies, i.e., the motivation to remove marks from their bodies but also their general attentiveness to markings, as in infants the ability to notice a change in a stimulus template (“Reizvorlage”) had a positive correlation with infants’ reactions to a mark on their face (Lewis and Brooks‐Gunn 1979). In the visible mark-controls, the birds were observed for a total of 5 min per session. The marks (sham and coloured) applied were the same as in the mark test. The choice in mark colour used in the ravens was based on Vanhooland et al.’s (2020) finding on the crows’ equal reactivity to red and blue mark in the visible mark control. For consistency reasons, we here only applied red marks to the ravens as these appeared more conspicuous on their plumage. We could, in these visible mark control sessions, post hoc confirm that the ravens indeed reacted strongly to these red markings (see “Results”), thus confirming the colour choice. However, in an attempt to improve on the original design, in the azure-winged magpies, this control was performed before and after the test. Marks were applied to the birds’ bellies and the reaction to the coloured mark in the pre-test control determined the colour used in the test for these birds (see Table 2).

## Data analysis

All habituation and test sessions were video-recorded using a Canon Legria ﻿HFG25 CMOS Pro. In every session, we recorded the amount of time the individual spent in front of, and in close proximity to the apparatus. In instances where an individual did not approach the apparatus (not even to retrieve the baits) during the individual sessions, the session was discarded and repeated. Coded behaviors (see Supplement 1 for full ethogram and description of the behaviors as well as Supplement 2 for video examples of the described behaviors) were pooled into four main categories based on the categorization of behaviors according to the level of mirror understanding (Rochat 2003): (1) social behaviors like self-aggrandizing or threat displays, attacks of the apparatus and vocalizations; (2) explorative behaviors e.g. including pecks directed towards the wooden frame of the apparatus and the inserted boards, search behaviors, i.e., attempts to perceive what is behind the apparatus (see Supplement 3 for a detailed analysis of the sub-categories of explorative behaviors); (3) contingent behaviors, including peekaboo behaviors and stretching; and, (4) self-directed behaviors such as autopreening, scratching, shaking, or bristling. This categorization was largely supported by the PCA analysis conducted on the data collected during the mirror exploration stage of the experiment (see Supplementary material 3). In the mark test, we further recorded all mark-directed behaviors, i.e., attempts to reach the mark with their beaks or feet. All behaviors were coded using Solomon Coder beta (András Peter) and later analysed in R (Version 4.0.0, R Core Team, 2020). An interrater reliability conducted on 10% of the video material showed a high degree of reliability between the two raters (AS and LV). The average ICC (assessed by a two-way model on the agreement between the raters) was 0.911 with a 95%-confidence interval estimate from 0.895 to 0.924 (*F* = 21.3, *p* < 0.001).

For further analysis, we determined average rates per minute of exposure of each behavior for each individual and condition. Sessions in which the birds did not approach the apparatus were discarded from the analysis.

Intraspecies analysis on the effect of the test condition on the response to the apparatus was done by performing Friedman tests. For the interspecies comparisons, we performed Kruskal–Wallis tests within the mirror and wood condition (in which we were able to compare the performances of all three corvid species) and Mann–Whitney tests in the silver foil condition (in which only the azure-winged magpies and common ravens were tested). Post hoc tests were done by pairwise comparisons. All reported *p* values from these post hoc tests have been adjusted using a Holm–Bonferroni correction (Holm 1979).

## Results

### Mirror exposure

In the following section, we first report the results of the effect of the test condition within the two species examined in this study and subsequently compare their performances with the performances of the carrion crows previously tested in the same paradigm by Vanhooland and colleagues (2020).

#### a. Intraspecies performance

**Fig. 3:**
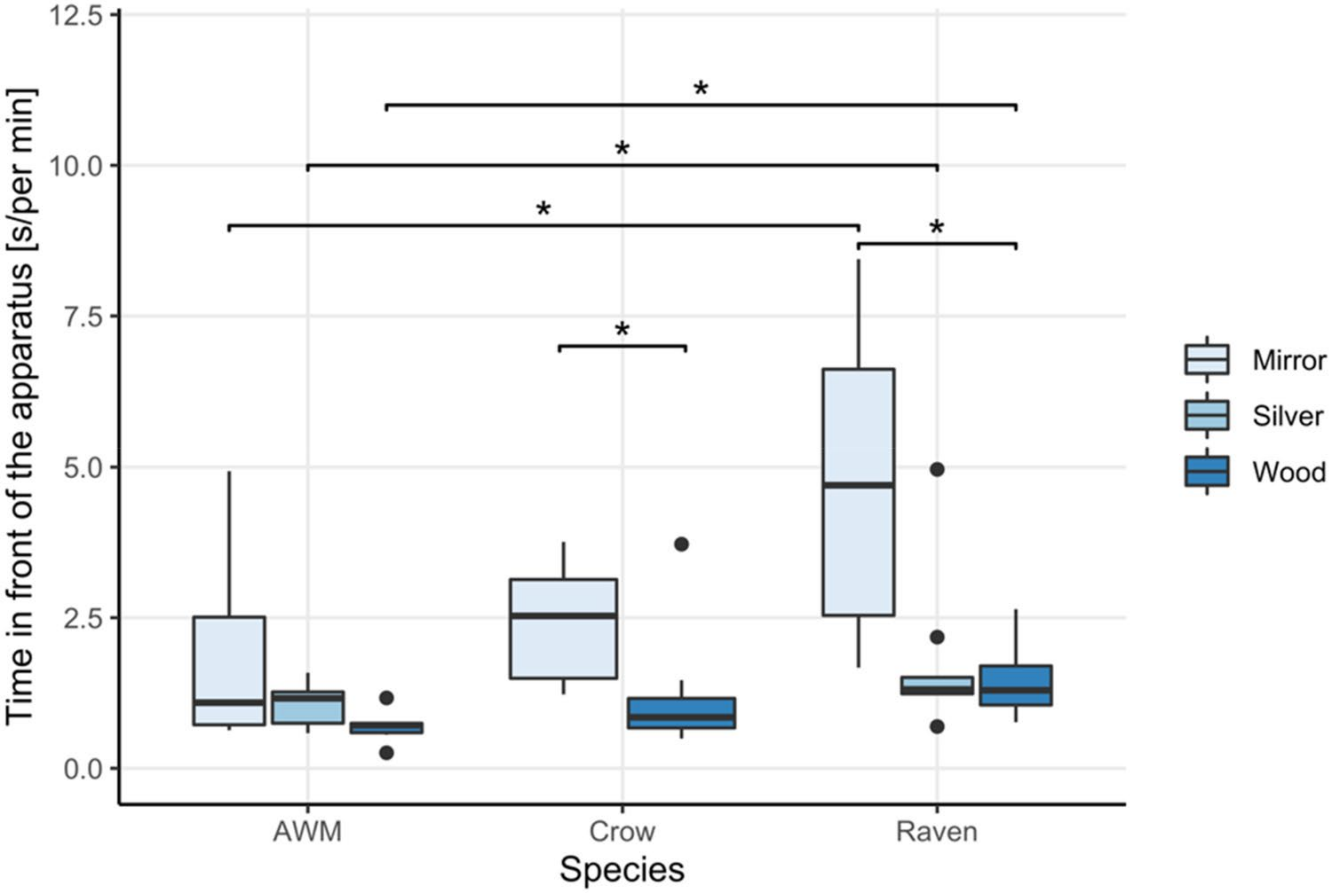
Average time spent in front of the apparatus (in seconds) by the three tested species in the test conditions of the mirror exposure phase (mirror, wood, and silver foil for the ravens and azure-winged magpies (AWM); mirror and wood for the carrion crows)

*Time spent at and in front of the apparatus.* During the mirror image stimulation phase of the experiment, condition had a significant effect on the time the ravens spent in close proximity of the apparatus (*χ*^2^ = 7.8, *df* = 2, *p* = 0.020) and more importantly in front of the apparatus (*χ*^2^ = 10.4, *df* = 2, *p* = 0.008). The ravens spent significantly more time around and in front of the apparatus when it contained the mirror as opposed to the wooden board (Time at apparatus_Mirror-Wood_: *V* = 52, *p* = 0.029; Time in front_Mirror-Wood_: *V* = 54, *p* = 0.012) as well as a tendency to spend less time with the silver foil (Time at apparatus_Mirror-Silver_: *V* = 48, *p* = 0.074; Time in front_Mirror-Silver_: *V* = 52, *p* = 0.055), while they did not favour the silver foil over the wooden board or vice versa (Time in front_Wood-Silver_: *V* = 31, *p* = 0.770; Time at apparatus_Wood-Silver_: *V* = 35, *p* = 0.492) (Fig. 3).

For the azure-winged magpies, condition had no effect on the amount of time the birds spent in front of the apparatus (*χ*^2^ = 4.33, *df* = 2, *p* = 0.115), but affected the time the birds spent in close proximity of the apparatus (*χ*^2^ = 6.33, *df* = 2, *p* = 0.042). However, contrarily to the ravens, the azure-winged magpies did not spend more time in front of the mirror than the wooden board (*V* = 20; *p* = 0.125). They further showed no significant difference between the silver foil and mirror (*V* = 21, *p* = 0.094) nor the silver foil and wood condition (*V* = 10, *p* = 1) (Fig. 3).

**Fig. 4:**
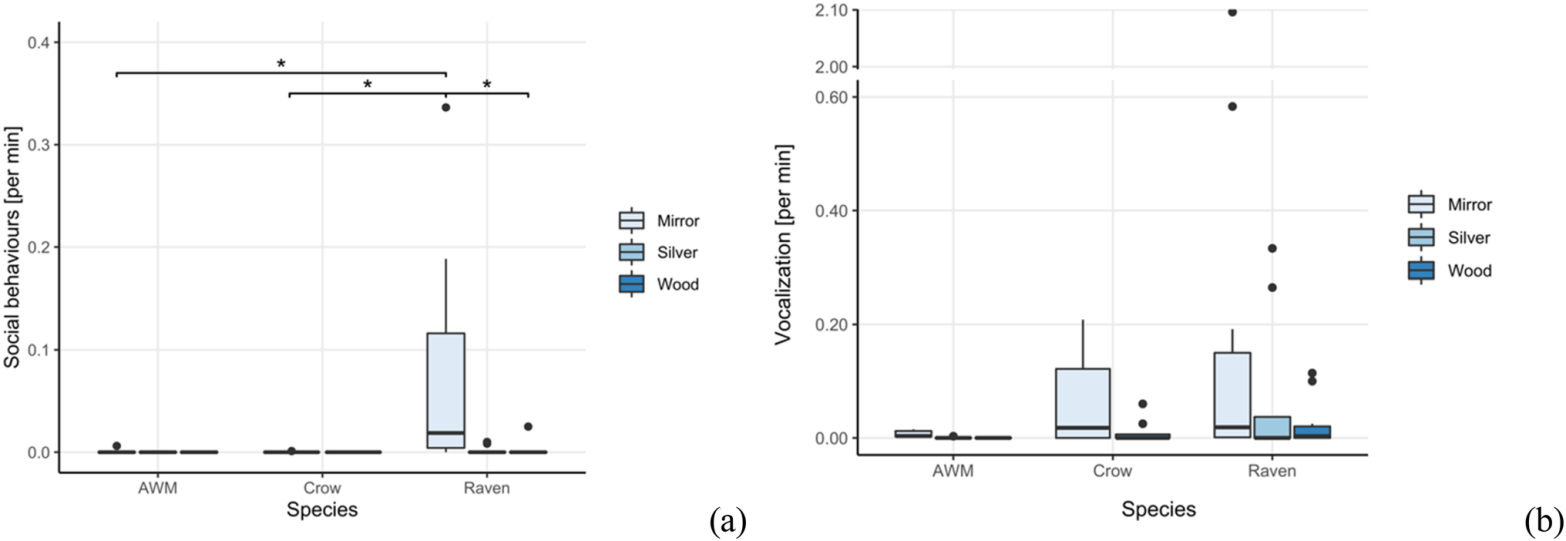
Average rate per minute of exposure of social behaviors (**a**) and vocalizations (**b**) exhibited by all three species in the different conditions of the mirror exposure phase of the experiment

*Social behaviors.* The test condition did affect the ravens’ expression of social behaviors (*χ*^2^ = 11.4, *df* = 2, *p* = 0.003). Ravens exhibited significantly more social behaviors towards the mirror than towards the silver foil (*V* = 36, *p* = 0.042), yet no significant difference was observed between the wood and mirror condition (*V* = 33, *p* = 0.084) nor between the wood and silver foil was found (*V* = 3, *p* = 1) (Fig. 4a). Condition had, however, no effect on the number of vocalizations emitted by the ravens (*χ*^2^ = 3.56, *df* = 2, *p* = 0.169) (Fig. 4b).

Unlike the ravens, the expression of social behaviors in the azure-winged magpies (*χ*^2^ = 2, *df* = 2, *p* = 0.368, Fig. 4a) was not affected by the test condition. It did, however, affect their propensity to vocalize (*χ*^2^ = 10, *df* = 2, *p* = 0.007), as the magpies almost exclusively vocalized in front of the apparatus in the mirror condition, albeit that post hoc comparisons of the mirror condition with the other condition did not render significant differences (Vocalizations_Mirror-Wood_: *V* = 15, *p* = 0.120; Vocalizations_Mirror-Silver_: *V* = 15, *p* = 0.120, Vocalizations_Wood-Silver:_ *V* = 0, *p* = 1) (Fig. 4b).

**Fig. 5:**
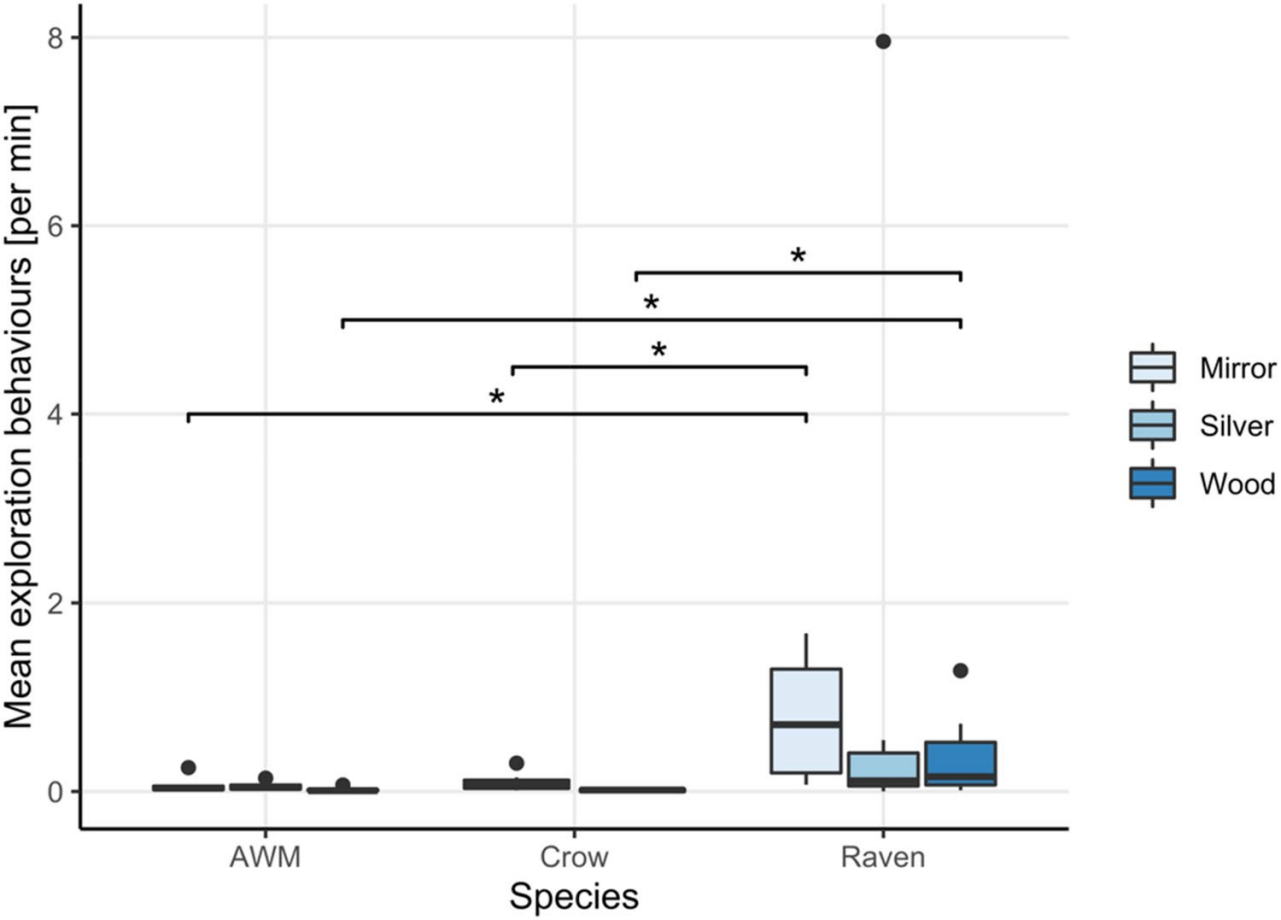
Average rate of exploration behaviors per minute exhibited by all three species in all conditions during the mirror exposure phase

*Exploration behaviors.* Exploration behaviors were the most commonly observed behavioral responses to the apparatus. We found that the ravens’ as well as the azure-winged magpies’ propensity to explore was affected by the test condition (exploration_Raven_: *χ*^2^ = 7.8, *df* = 2, *p* = 0.020; exploration_AWM_: *χ*^2^ = 7, *df* = 2, *p* = 0.030) yet no significant difference was found between conditions in post hoc pairwise comparisons (Ravens: exploration_Mirror-Wood_: *V* = 49, *p* = 0.082; exploration_Mirror-Silver_: *V* = 40, *p* = 0.465; exploration_Wood-Silver_: *V* = 22, *p* = 0.625; AWM: exploration_Mirror-Wood_: *V* = 16, *p* = 0.625; exploration_Mirror-Silver_: *V* = 8, *p* = 0.688; exploration_Wood-Silver_: *V* = 21, *p* = 0.094; Fig. 5).

**Fig. 6:**
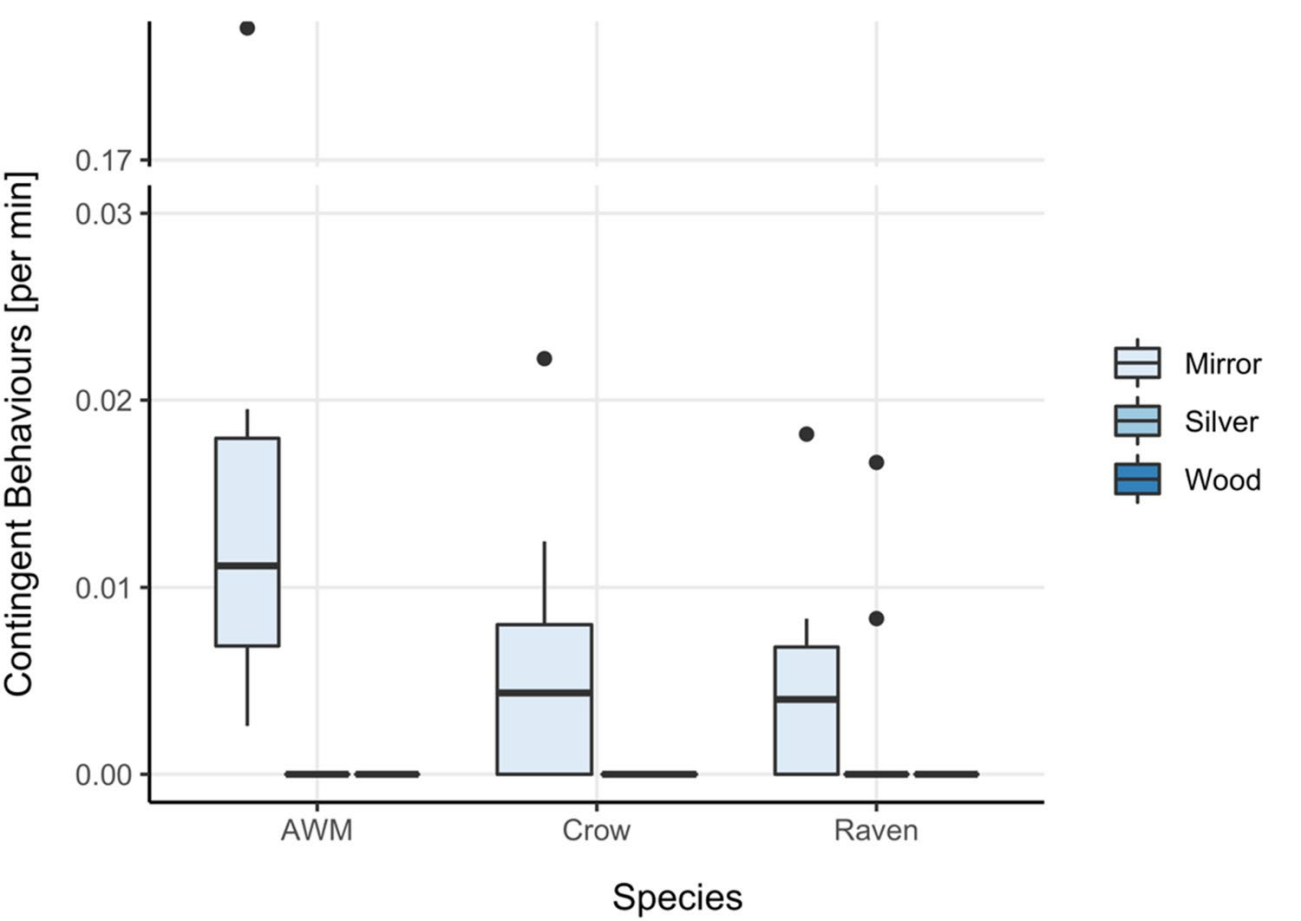
Average rate per minute of exhibited contingent behaviors during the mirror exposure phase

*Contingent behaviors.* The test conditions significantly affected the contingent behaviors of the ravens (*χ*^2^ = 8.818, *df* = 2, *p* = 0.012) and AWM (*χ*^2^ = 12, *df* = 2, *p* = 0.002). While post hoc contrasts revealed no significant difference between conditions in the ravens (contingent_Mirror-Wood_: *V* = 21, *p* = 0.110, contingent_Mirror-Silver_: *V* = 21, *p* = 0.540; contingent_Wood-Silver_: *V* = 3, *p* = 0.540; Fig. 6), the AWM showed a tendency to perform these behaviors more in the mirror condition (contingent_Mirror-Wood_: V = 21, p = 0.063, contingent_Mirror-Silver_: *V* = 21, *p* = 0.063; contingent_Wood-Silver_: *V* = 0, *p* = 1; Fig. 6).

**Fig. 7:**
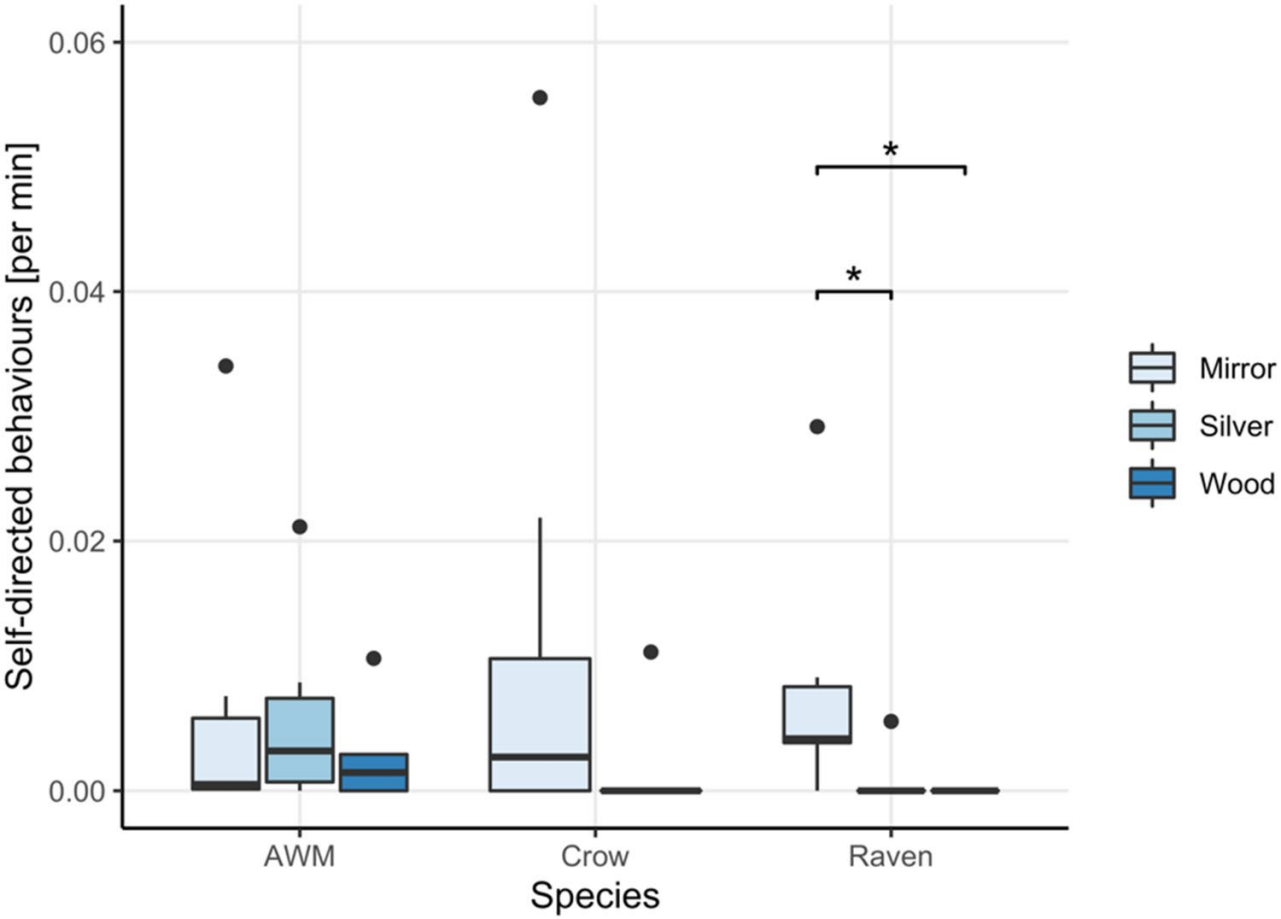
Average amount of exhibited self-directed behaviors during the mirror exposure phase in each of the three test conditions

*Self-directed behaviors.* The ravens’ self-directed behaviors were significantly influenced by the test condition (*χ*^2^ = 15.44, *df* = 2, *p* < 0.001; Fig. 7) as they exhibited significantly more self-directed behaviors in the mirror condition as compared to the silver foil (*V* = 36, *p* = 0.042) or wooden board condition (*V* = 36, *p* = 0.042), while there was no difference between the wood and silver conditions (*V* = 1, *p* = 1). The test condition did, however, not affect the expression of self-directed behaviors in the azure-winged magpies (*χ*^2^ = 3.5, *df* = 2, *p* = 0.172; Fig. 7).

#### b. Interspecies comparison

The performances of all three species were compared in the mirror and the wood condition. In the silver foil condition, comparisons were drawn between the raven and azure-winged magpies only, as the crows had not been exposed to the silver foil in their test sessions in Vanhooland et al. (2020).

*Durations.* We found a significant species effect on the time spent in front of the apparatus for each of the three test conditions (Mirror: *χ*^2^ = 8.4, *df* = 2, *p* = 0.015; Wood: *χ*^2^ = 10.1, *df* = 2, *p* = 0.006 and Silver foil: *W* = 11, *p* = 0.041). The ravens spent significantly more time in front of the apparatus than the azure-winged magpies in all three conditions (post hoc pairwise comparison after Holm–Bonferroni correction: Raven-AWM_Mirror_: *W* = 8, *p* = 0.048; Raven-AWM_Wood_: *W* = 2; *p* = 0.003; Raven-AWM_Silver_: *W* = 11, *p* = 0.041). The ravens also showed a tendency to spend more time in front of the apparatus than the crows in the mirror but not the wood condition (Raven-Crow_Mirror_: *W* = 15, *p* = 0.053; Raven-Crow_Wood_: *W* = 21, *p* = 0.101). We found no difference in the time spent in front of the apparatus between the crows and azure-winged magpies (Mirror: *W* = 14, *p* = 0.228; Wood: *W* = 11, *p* = 0.108). We further found a species effect on the time spent around the apparatus for the mirror (*χ*^2^ = 12.2, *df* = 2, *p* = 0.002) and wood (*χ*^2^ = 9.9, *df* = 2, *p* = 0.007) but not the silver foil condition (*W* = 20, *p* = 0.313). The crows spent significantly less time around the apparatus than the ravens in the mirror (Raven-Crow: *W* = 4, *p* = 0.002) and the wood condition (Raven-Crow: *W* = 7, *p* = 0.006). Compared to the AWM, the crows showed a tendency to spend more time around the apparatus in the mirror condition (AWM-Crow_Mirror_: *W* = 41, *p* = 0.059) and significantly more time in the wood condition (AWM-Crow_Wood_: *W* = 42, *p* = 0.040), while no differences were found between the amount of time spent around the apparatus between the ravens and azure-winged magpies (Mirror: Raven-AWM: *W* = 15, *p* = 0.118; Wood: Raven-AWM: *W* = 25, *p* = 0.635; Silver foil: *W* = 20, *p* = 0.313).

*Social behaviors.* There were no interspecies differences in the number of vocalizations emitted in any of the conditions (Silver: *W* = 18, *p* = 0.101; Wood: *χ*^2^ = 4.2, *df* = 2, *p* = 0.123; Mirror: *χ*^2^ = 1.18, *df* = 2, *p* = 0.556). We did, however, find a significant interspecies difference in the expression of social behavior in front of the mirror (*χ*^2^ = 11.62, *df* = 2, *p* = 0.003), but no differences in wood (*χ*^2^ = 1.4, *df* = 2, *p* = 0.497) or silver condition (*W* = 24, *p* = 0.299). The ravens performed more social behaviors in front of the mirror than the azure-winged magpies and the carrion crows (Raven-AWM: *W* = 9, *p* = 0.040, Raven-Crow: *W* = 9, *p* = 0.011), while no differences were found between the crows and the azure-winged magpies (*W* = 25.5, *p* = 0.832).

*Exploration behaviors.* We found significant interspecies difference in the exploration behaviors in the mirror (*χ*^2^ = 12.258, *df* = 2, *p* = 0.002), wood (*χ*^2^ = 10.998, *df* = 2, *p* = 0.004) but not in the silver foil condition (*W* = 16, *p* = 0.147). The ravens exhibited significantly more exploration behaviors towards the mirror and the wooden board than the other two species (Mirror Exploration_Raven-Crow_: *W* = 9, *p* = 0.009; Mirror Exploration_Raven-AWM_: *W* = 3, *p* = 0.005; Mirror Exploration_Crow-AWM_: *W* = 15; *p* = 0.2824; Wood Exploration_Raven-Crow_: *W* = 9, *p* = 0.014; Wood Exploration_Raven-AWM_: *W* = 3, *p* = 0.014), but we found no significant differences between the exploration behaviors of the crows and azure-winged magpies (Mirror Exploration_AWM-Crow_: *W* = 15, *p* = 0.282; Wood Exploration_AWM-Crow_: *W* = 15, *p* = 1).

*Contingent behaviors.* We found no significant interspecies differences in the exhibition of contingency checking behaviors in any of the three test conditions. None of the birds performed contingency checking behaviors in the wood condition, nor did the species perform differently in the mirror *χ*^2^ = 4.598, *df* = 2, *p* = 0.100) or silver foil (*W* = 24, *p* = 0.300) condition.

*Self-directed behaviors.* The three species showed no significant differences in their self-directed behaviors in the mirror (*χ*^2^ = 0.71, *df* = 2, *p* = 0.700) nor the wood (*χ*^2^ = 1.53, *df* = 2, *p* = 0.466) condition. The azure-winged magpies did however exhibit significantly more self-directed behaviors than the ravens in the silver foil condition (*W* = 49, *p* = 0.015).

### Mark test

**Fig. 8:**
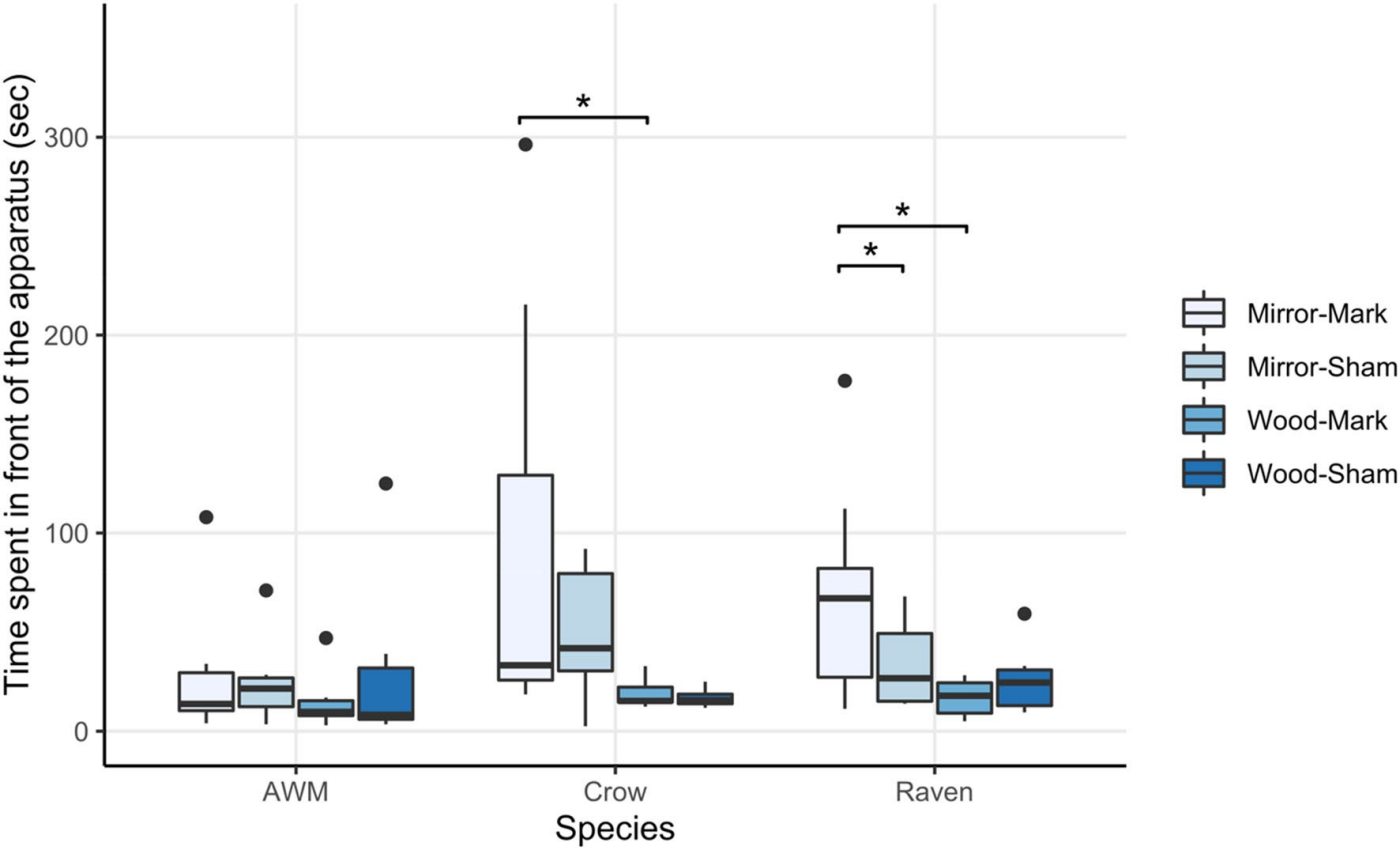
Average time spent in front of the apparatus by all three corvid species in the mirror-mark, mirror-sham, wood-mark, and wood-sham conditions of the mark test

To evaluate the performances of the birds in the mark test, we examined the amount of time the individuals spend in front of the apparatus in each treatment condition (Fig. 8) as well as the number of mark-directed and self-directed behaviors they performed while standing in front of the apparatus.

#### a. Ravens

The treatment condition had a significant effect on the time the ravens spent in front of the apparatus (*χ*^2^ = 10.92, *df* = 3, *p* = 0.012) as they stayed significantly longer in front of the apparatus in the mirror-mark condition than the mirror-sham (*V* = 51, *p* = 0.041) and wood-mark condition (*V* = 54, *p* = 0.012), while there was no difference between the mirror-sham and wood-sham condition (*V* = 39, *p* = 0.826). Yet, none of the ravens showed any mark-directed behaviors in the test sessions.

#### b. AWM

The azure-winged magpies did not spend significantly more time in front of the apparatus in any of the test conditions (*χ*^2^ = 4.808, *df* = 5, *p* = 0.4398) nor did any of them exhibit mark-directed behaviors.

#### c. Interspecies comparison

There were no interspecies differences within the four common treatment conditions (Mirror-Mark: *χ*^2^ = 3.57, *df* = 2, *p* = 0.168; Mirror-Sham: *χ*^2^ = 3.22, *df* = 2, *p* = 0.199; Wood-Mark: *χ*^2^ = 1.82, *df* = 2, *p* = 0.403; Wood-Sham: *χ*^2^ = 1.49, *df* = 2, *p* = 0.476).

#### d. Visible mark control

Both species reacted significantly more to the visible coloured marks than the sham mark when placed on visible body parts. They showed more frequent (*W* = 432.5, *p* < 0.001) and longer (*W* = 367, *p* < 0.001) mark-directed behaviors towards the coloured mark, while similar behaviors were almost never observed in the sham mark condition. Most responses to the coloured mark happened within the minute after the marking, average response latency to the coloured mark: 31.8 ± 23.7 s. Because not all azure-winged magpies reacted to the visible red mark in the pre-test session, other colours (green and yellow) were used to entice the bird’s reaction. Out of the 6 birds, 3 reacted to all 3 colours, 2 reacted only to the red and yellow mark and one exclusively to the yellow markings. We further did not find any significant difference between the propensity to respond to the coloured marks between the pre- and post-test in the azure-winged magpies (*χ*^2^ = 1.06, *df* = 1, *p* = 0.304).

## Discussion

In this study, we first explored the mirror responses and abilities to pass the mark test of two corvid species: the common raven and the azure-winged magpie and second compared the performances of these two species with each other and a third corvid species, previously tested following a comparable procedure: the carrion crow (Vanhooland et al. 2020). We found that in the mirror exposure phase of the experiment (in which the ravens and azure-winged magpies were exposed to either a mirror, a wooden board, or a silver foil in the apparatus), only ravens showed a clear preference for the mirror compared to the other surfaces and performed more self-directed behaviors when in front of the mirror. Contrarily to expectations, the AMW did not behave differently in front of the mirror than in the other test conditions and were less explorative than the ravens. Comparatively, the ravens spent significantly more time in front of the mirror than the two other species and, as predicted, had a stronger agonistic social response towards the mirror than the AWM and crows. They further exhibited overall more explorative behaviors towards the mirror and apparatus. In the mark test, again in contrast to the other two species, ravens spent significantly more time in front of the mirror when marked with a coloured mark then in any other test condition which could indicate that the ravens did perceive a difference in the mirror image. Yet, none of the species showed mark-directed behaviors indicative of mirror self-recognition, although all individuals were motivated to remove coloured marks from their bodies when they could observe these markings without the use of a mirror.

When exploring the effect of the test conditions (i.e., mirror, wood, or silver foil) on the birds’ behaviors, we found that similarly to the carrion crows (Vanhooland et al. 2020), common ravens exhibited a clear preference for the mirror. We further observed that both the ravens and the azure-winged magpies did not exhibit a particular interest in the silver foil over the wooden board. The ravens’ preference for the mirror over the silver foil therefore indicates that the preference for mirrors is not only the result of the objects novelty or shininess but rather results from the mirror’s inherent reflective properties. A preference, contrarily to our expectations, is not shared by the AWM.

Overall, the azure-winged magpies spent much less time at the apparatus in a given session and therefore, on average, received more test sessions than the ravens and the crows (to reach the set exposure criterium). They also spent considerably less time in front of the mirror than the azure-winged magpies of Wang and colleagues (2020) who reported that the azure-winged magpies that entered the test compartment spent 27–47% of their time in front of the mirror and 2–8% in front of the none-reflective control when given the choice. In comparison, during the first five sessions, the azure-winged magpies in our study spent 0.65–5.6% and 0.25–0.71% of their time in front of the mirror and the wooden board, respectively, yet were faster at approaching the apparatus (all magpies approached the apparatus in their first session, compared to only half of the subjects in Wang et al. (2020)). An explanation for the lower interaction durations with the apparatus could be the magpies’ neophobic reaction towards the apparatus. Indeed, corvids are known to be species that score higher on the neophobic scale. Neophobia is also known to be a big confound in cognitive tasks. Yet, in a recent large-scale comparative study on neophobia in corvids (Miller et al. 2022) azure-winged magpies reached lower scores of object neophobia than the carrion crows and common ravens. Therefore, the azure-winged magpies’ comparatively lower interest in the apparatus does not likely seem to be explained by their higher neophobia. Further, the differences observed with the azure-winged magpies from Wang et al. (2020) could at least partially be explained by the difference in experimental setup, as the azure-winged magpies in the Wang et al. (2020) study were offered a choice task in which both the mirror and non-reflective board were presented at the same time in the bird’s test compartment, while the birds in our study were only presented with one condition per session. The azure-winged magpies in Wang and colleagues (2020) study were further tested in a considerably smaller experimental compartment (dimensions: 60 × 40 × 40cm vs. 2.25 × 3 × 3m) giving the birds fewer alternative occupations (e.g., caching, pilfering) besides the interaction with the apparatus. Finally, the age and the housing of the birds might further account for some of the differences observed between the studies, as the subjects tested by Wang et al. (2020) were singly housed juveniles. Younger individuals are often observed to be more explorative than their adult counterparts (Biondi et al. 2013; Greggor et al. 2020) and singly housed individuals might be more receptive for the social feedback given by the mirror (Henry et al. 2008) than socially housed individuals like the ones in our sample.

Similar to the carrion crows (Vanhooland et al. 2020), the behaviors exhibited by the ravens and azure-winged magpies throughout the study do not appear indicative of a state of complete lack of awareness (i.e., Rochat’s level 0 of confusing between the reflections in the mirror and the environment). Yet, more investigations would be necessary to more clearly determine these species level of mirror understanding. In particular, because neither the azure-winged magpie nor the common ravens showed statistically significant differences between the conditions in their contingency checking behaviors, despite occurring predominantly in front of the mirror. Contingency checking behaviors during mirror exploration are defined as behaviors directed towards the mirror that individuals use to test the correspondence between their own movement and the movement observed in the mirror, and which are commonly of a repetitive or unusual nature. Although the birds in this study exhibited behaviors that could be deemed consistent with the test of correspondence between own movements and the movement of the reflection (mainly peekaboo behaviors that were expressed very similarly across the three corvid species tested in this study, see Supplement 2), these behaviors were not seen to be performed repetitively, as commonly observed in transition phases of self-recognizing species [e.g., dolphins (Reiss and Marino 2001), elephants (Plotnik et al. 2006), or chimpanzees (Povinelli et al. 1993)]. It is conceivable that such behaviors might be subject to interspecies variations, thus, highlighting the importance of careful reporting of the working definitions used for the categorization and interpretation of observed behaviors. Contrarily, it is also possible that the behaviors observed in these corvids do not reflect the same level of mirror understanding as the repetitive behaviors observed in self-recognizing species, in which contingent behaviors are considered precursors for self-directed behaviors that reportedly arise shortly before the expression of mirror self-recognition in the ontogenetic development, at least in humans and chimpanzees (Lin et al. 1992; Povinelli et al. 1993). Interestingly, the common ravens still exhibited significantly more self-directed behaviors in the mirror than the control conditions. The exhibition of self-directed behaviors is considered the first indication of an individual’s ability to recognize itself in a mirror (Gallup 1970; Povinelli et al. 1993). In primates, those behaviors are commonly associated with the exploration of body parts the individual would not be able to perceive without the use of a mirror (e.g. the eyes, the inside of their mouth). However, based on the avian visual fields (Hart and Scassellati 2012), we can assume that in ravens, such body parts are few (i.e., restricted to some areas of the head and the inside of their beaks). The self-directed behaviors exhibited by the ravens in our study are thus more difficult to interpret, and might not have the same standing and meaning as the ones commonly observed in great apes. Indeed, in birds, like in mammals, increased preening behaviors have also been observed to function as a coping behavior in a stressful situation (Henson et al. 2012). The increase in self-directed behaviors could therefore also reflect elevated arousal levels as to be expected in the mirror condition. This emphasizes the importance of testing corvids in situations with as little added stress as possible to avoid interferences. This becomes particularly relevant in the mark test (where such increased stress resulting in increased self-directed behaviors can lead to increased accidental mark-directed behaviors and thus potential false positives), and should be kept in mind in the pre-test manipulations of the birds, particularly given the fact that, in contrast to our study, catching and restraining birds for marking are still the norm when testing avian species (Table 1).

Despite the exhibition of self-directed behaviors in the mirror condition by the ravens, neither the ravens nor the azure-winged magpies, as the carrion crows before them, exhibited any mark-directed behaviors during the mark test. This failure of the mark test is in line with the performances of many other corvid species (Soler et al. 2014, 2020; Clary and Kelly 2016; Brecht et al. 2020; Clary et al. 2020; Smirnova et al. 2020; Vanhooland et al. 2020; Wang et al. 2020; Parishar et al. 2021). Our results further replicate and confirm the previous findings of azure-winged magpies failing the mark test and not exhibiting mark-directed behaviors when the mark can only be seen by utilizing a mirror (Wang et al. 2020).

The azure-winged magpies, carrion crows, and ravens, however, interestingly differed in the amount of time they spend in front of the apparatus in each of the four conditions of the mark test. While carrion crows (Vanhooland et al. 2020) had been shown to spend more time in front of the apparatus in the mirror conditions than in the wood conditions of the mark test (in line with their behaviors in the mirror exposure phase of the study), their behaviors were not affected by the type of mark applied to the them. Similarly in line with their previous performances in the exposure phase, neither the condition (mirror or wood) nor the type of marking (colour or sham) had an effect on the magpies’ time spent in front of the apparatus. Per contra, the ravens’ time spent in front of the apparatus was not only increased by the presence of the mirror but also by the presence of a coloured mark, in contrast to a sham mark, on them in the mirror condition. While this does not provide evidence of self-recognition in the ravens, it does indicate that the ravens perceived a difference in their reflection between the coloured and sham marking, which may be the result of an expectancy violation. Yet, whether it violated the expectancy of the representation the bird had of its own body image or of the representation of a conspecific, and whether ravens therefore have a concept of self that the mark test was not sensitive enough to determine, remains to be determined.

Thus far, whether the interspecies and interindividual differences in observed mark test performances of corvids result from phylogenetic, ecological, cognitive, or methodological differences, which have all been shown to affect the results of the mark test, remains uncertain. In spite of still being the go-to test for investigations of self-recognition and self-awareness in non-human animals, the mark test has been the object of criticism due to the results it generates, their interpretation, and the use of this test as a stand-alone method (De Veer and van den Bos 1999; Bard et al. 2006; Heschl and Burkart 2006). The results from the mark test generally present a substantial within species variation and a low success rate (Povinelli et al. 1993; Keller et al. 2005). These within-species variations are not restricted to non-human animals; in fact, variations in children have been attributed to factors such as cultural variations (Broesch et al. 2011; Ross et al. 2016), parenting styles (Keller et al. 2005), and mother–infant attachment (Lewis et al. 1985). Recent studies further demonstrated the effect of an individual’s genotype (Mahovetz et al. 2016) and neuroanatomy (Hecht et al. 2017; Hopkins et al. 2019) on the MSR performances in chimpanzees. Furthermore, the validity of mirror self-recognition (in the form of passing the mark test) as an indicator of self-awareness has been questioned and several alternative interpretations proposed (Schilhab 2004; Suddendorf and Butler 2013). This still predominant theoretical construct, calling on richer interpretations, is further being challenged by findings of fish (Kohda et al. 2019, 2022) and ants (Cammaerts and Cammaerts 2015) passing the mark test. Finally, while the interspecies differences observed in primates seem clearly driven by phylogeny and the result of a divergent evolution between great apes and monkeys (Anderson et al. 2011; Anderson and Gallup 2015), and the cases of convergent evolution seem to be driven by factors such as high encephalization, the high complexity of the species’ social system, and the evincing of advanced cognitive abilities (Reiss and Marino 2001; Plotnik et al. 2006), these explanations do not seem to be sufficient to explain the differences in performances observed within the corvid taxa. This underlines the necessity for more large-scale comparative studies, like the current study, exploring the effects of methodological and ecological factors on the responses to mirrors in corvids as well as the necessity for more in-depth studies on the cognitive abilities of these species in MSR-related domains to explore the cognitive characteristics associated with MSR in corvids.

Such comparative investigations further benefit the construct of a gradualist approach of self-awareness (de Waal 2019) currently not supported by the pass-or-fail outcome of the mark test, which on the one hand might lack the sensitivity to detect more subtle differences in performance and on the other hand promotes a misleading impression that one can either be fully self-aware or not possess any self-awareness at all, as opposed to being situated on a continuum of the self-awareness spectrum (de Waal 2019; Baciadonna et al. 2021). Future studies might thus benefit from including measures that more systematically evaluate different levels of mirror understanding and self-awareness, which could result in a categorization of species beyond self-recognizing versus non-self-recognizing. Such measures could include the comparison of an individual’s responses to mirrors versus their response to familiar and unfamiliar conspecifics as to determine whether the individuals are on the level of mirror confusion (as defined by Rochat (2003)) or measures indicating the understanding of a correspondence between the reflection and the environment by showing the ability to use the mirror to for example locate an object out of the individual’s direct line of sight (Ünver et al. 2017).

Finally, there is an inherent problem in studying the broad concept of self-awareness in different taxonomic groups by implementing a single test designed for species with hands and mammalian visual systems, that solely addresses one facet of self-awareness (Parker et al. 1994; de Waal 2019; Baciadonna et al. 2021). Future investigations into self-recognition and self-awareness will therefore require the development of new paradigms and a truly comparative approach to allow a more diverse assessment of what constitutes self-awareness.

## Supplementary Information

Below is the link to the electronic supplementary material.

Supplementary file1 (MP4 115724 KB)

Supplementary file2 (MP4 76848 KB)

Supplementary file3 (MP4 37802 KB)

Supplementary file4 (MP4 100522 KB)

Supplementary file5 (DOCX 6205 KB)
