# Supplementary material for: A comparative study of mirror self-recognition in three corvid species
Source: Anim Cogn. 2022 Sep 29;26(1):229–48. doi: 10.1007/s10071-022-01696-4 (PMC9876878; doi:10.1007/s10071-022-01696-4)
Supplement: Supplementary file 5 — Supplementary file5 (DOCX 6205 KB) [file 10071_2022_1696_MOESM5_ESM.docx]

**Supplementary material**

**Supplement 1. Ethogram**

Table S1. Ethogram of mirror-related behaviours exhibited by each species

| Social behaviours | Vocalizations | | Bird vocalizes while in front of the apparatus |
| --- | --- | --- | --- |
|  | Attack | | Bird displays aggressive and fighting behaviours towards the apparatus while standing in front of it |
|  | Self-aggrandizing displays | | Bird exhibits dominance or threat displays while in front of the apparatus |
| Exploration behaviours | Surface exploration | Bite surface | Bird attempts to bite the plate inserted into the apparatus |
|  |  | Peck surface | Bird pecks the plate inserted into the apparatus |
|  |  | Clawing | Bird scratches the apparatus with its foot |
|  | Frame exploration | Bite frame | Bird bites into the apparatus frames |
|  |  | Peck frame | Bird pecks the apparatus frame |
|  | Search behaviours | Look under | Bird looks under the apparatus. |
|  |  | Look behind | Bird looks behind the apparatus |
|  | Look down | | Bird is perched on top of the apparatus and looks down into the mirror; usually rapidly moving in and out of sight of the mirror |
| Contingent behaviours | Peekaboo | | Bird moves in and out of view of the inserted plate within 5 seconds (i.e. past the frame of the apparatus) |
|  | Stretch | | Bird stretches its leg or wing |
| Self-directed behaviours | Shake | | Bird shakes in front of the apparatus |
|  | Auto-preening | | Bird cleans its plumage |
|  | Scratch | | Bird scratches itself (with its foot) |
|  | Bristle | | Bird shortly lifts its feathers |
|  | Head shake | | Bird shakes its head |
| Location | Time around apparatus | | Time the bird spends in close proximity (~2 body lengths) of the apparatus while next to, behind or perched on top of the apparatus |
|  | Time in front | | Time the bird spends in front of the apparatus (up to 2 body lengths away) |

**Supplement 2. Videos of behaviours exhibited by the azure-winged magpies and common ravens**

- **Video 1: Social behaviours**
- **Video 2: Exploration behaviours**
- **Video 3: Contingent behaviours**
- **Video 4: Self-directed behaviours**

**Supplement 3. Detailed analysis of different types of exploration behaviours exhibited towards the apparatus**

1. *Data Analysis*

In light of the results of the PCA analysis which classified the sub-categories of exploration behaviours in three different components we conducted a more in-depth analysis of the explorative behaviours exhibited by the birds distinguishing between exploration behaviours directed toward the wooden frame of the apparatus (i.e. frame exploration), directed towards the inserted boards (i.e. surface exploration), attempts to perceive what is behind the apparatus (i.e. searching behaviour including looks behind the apparatus and looks under the apparatus) and looks down towards the surface while the bird is perched on top of the apparatus (i.e. look down). In line with the analysis conducted for the other behavioural categories presented, we determined averages rates per minute of exposure of each behaviour for each individual and condition. Intraspecies analysis on the effect of the test condition on the response to the apparatus were done by performing Friedman-tests. For the interspecies comparisons, we performed Kruskal-Wallis tests within the mirror and wood condition (in which we were able to compare the performances of all three corvid species) and Mann-Whitney tests in the silver foil condition (in which only the azure-winged magpies and common ravens were tested). Post-hoc tests were done by pairwise comparisons. All reported p-values from these post-hoc tests have been adjusted using a Holm-Bonferroni correction(Holm 1979).

1. *Results*
2. Intraspecies performance

We further differentiated between the exploration of the different surfaces inserted into the apparatus ( i.e. the mirror, silver foil and wood), the exploration of the wooden frame of the apparatus, searching behaviours (i.e. attempts to look behind the apparatus) and look down behaviours (when the bird was perched on top of the apparatus and bowed down to reach the surface of the apparatus) (Figure S1). In the ravens, none of these behaviours were affected by the test condition (surface exploration: χ^2^=5, df=2, p=0.082; frame exploration: χ^2^=4.1, df=2, p=0.132; search behaviours: χ^2^= 3.4, df=2, p=0.179; look down: χ^2^=3.846, df=2, p=0.146; Figure S1). In the azure-winged magpies the test condition significantly affected their frame exploration (χ^2^=7.6, df=2, p=0.022), their search behaviours (χ^2^=8.1, df=2, p=0.018) as well as their look down behaviours (χ^2^=8.435, df=2, p=0.015)*,* yet we found no significant differences between conditions in the post-hoc pairwise comparisons (frame exploration_Mirror-Wood_: V=0, p=0.177, frame exploration_Mirror-Silver_: V=0, p=0.177, frame exploration_Wood-Silver_: V=12, p=0.280; search_Mirror-Wood_: V=21, p=0.094, search_Mirror-Silver_: V=9, p=0.844, search_Wood-Silver_: V=15, p=0.177; look down_Mirror-Wood_: V=21, p=0.094, look down_Mirror-Silver_: V=16, p=0.313, look down_Wood-Silver_: V=15, p=0.118; Figure S1). Condition had no effect on the azure-winged magpies´ surface exploration (χ^2^=0.3, df=2, p=0.869) (Figure S1).

1. Interspecies comparisons

More precisely, we found significant interspecies differences in frame and surface exploration behaviours as well as in the looks down in the mirror condition (frame: χ^2^=16.87, df=2, p< 0.001; surface: χ^2^=6.77, df=2, p=0.034; look down: χ^2^=13.553, df=2, p=0.011; Figure S1) as well as in the wood condition (frame: χ^2^=13.03, df=2, p=0.002; surface: χ^2^=7.71, df=2, p=0.021; look down: χ^2^=7.866, df=2, p=0.020; Figure S1) but not in the silver foil condition (frame: W=25.5, p=0.662; surface: W=14, p=0.088; look down: W=22.5, p=0.444, Figure S1). In the mirror condition, the ravens exhibited significantly more frame and surface exploration behaviours than the azure-winged magpies (post-hoc pairwise comparisons: frame _Raven-AWM_: W=0, p=0.003; surface _Raven-AWM_: W=6, p=0.022) as well as performing significantly more frame but not surface exploration behaviours then the crows (frame_Raven-Crow_: W=2, p=0.003; surface _Raven-Crow_: W=30, p=0.408). We found no differences in frame nor surface exploration behaviours between the crows and azure-winged magpies in the mirror condition (frame_Crow-AWM_: W=13.5, p=0.179; surface_Crow-AWM_: W=11.5, p=0.242, Figure S1). The azure-winged magpies and ravens further exhibited significantly more look down behaviours than the crows in the mirror condition (look down_Crow-AWM_: W=44, p=0.029; look down_Raven-Crow_: W=2, p=0.002), but we found no difference between the AWM and the ravens (look down_Raven-AWM_: W=19, p=0.251) (Figure S1). In the wood condition, the ravens explored the frame of the apparatus as well as the surface of the apparatus more than the crows but not the azure-winged magpies (frame_Raven-AWM_: W=11.5, p=0.057; frame_Raven-Crow_: W=8, p=0.006; surface_Raven-Crow_: W=16, p=0.039; surface_Raven-AWM_: W=18, p=0.193; Figure S1). In addition, the azure-winged magpies showed more frame exploration (W=44, p=0.007) but not more surface exploration (W = 36, p=0.078) than the crows in the wood condition. Pairwise post-hoc comparisons of the look down behaviour in the wood condition rendered no significant interspecies differences and just a tendency for the ravens to perform this behaviour more frequently than the crows (look down_Crow-AWM_: W=28, p=0.530; look down_Raven-Crow_: W=15.5, p=0.057, look down_Raven-AWM_: W=19, p=0.095, Figure S1). We further found no significant interspecies differences in the search behaviours in any of the conditions (mirror: χ^2^=1.59, df=2, p=0.451; wood: χ^2^=5.41, df=2, p=0.067; silver: W=47.5, p=0.058; Figure S1).


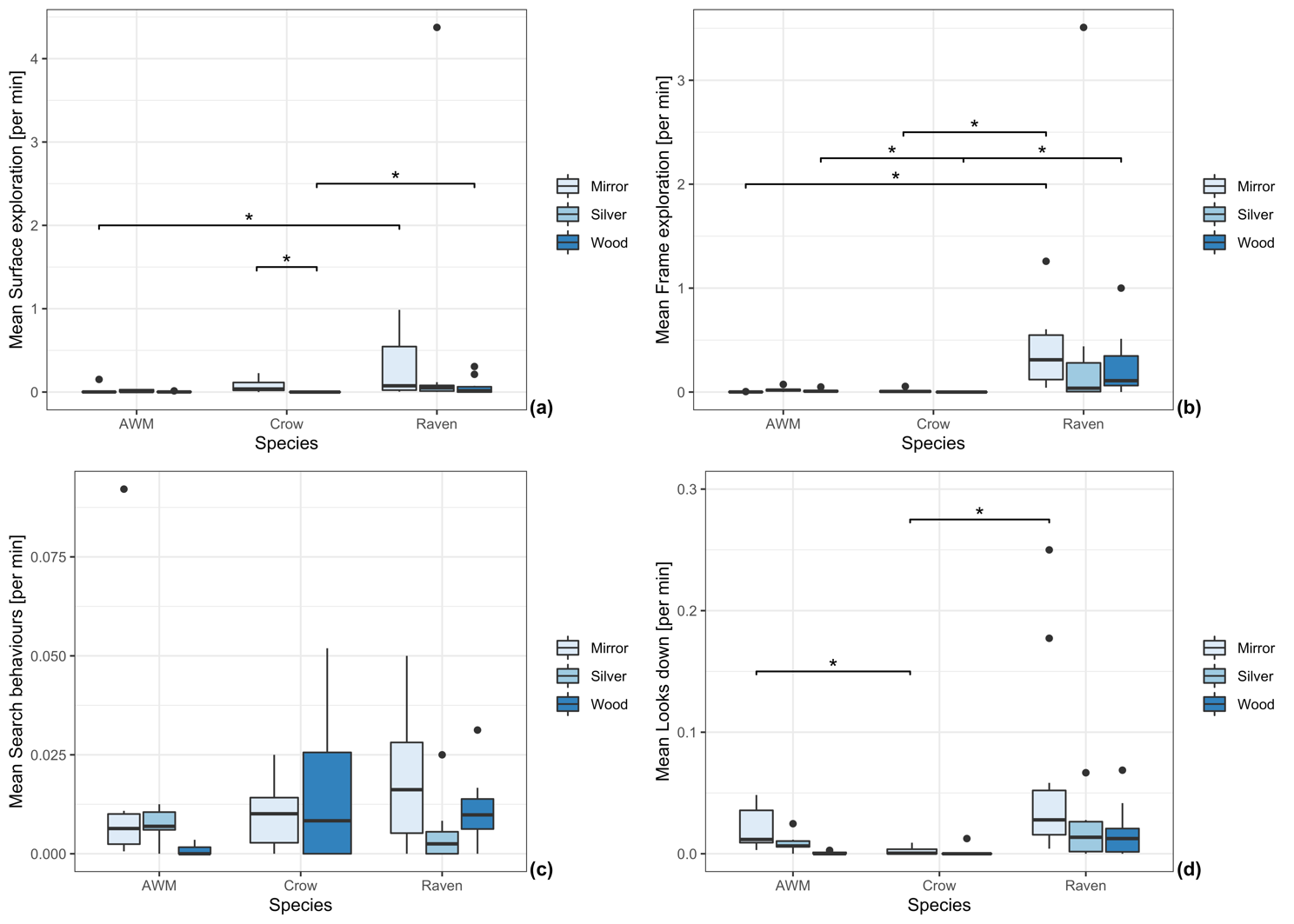


Figure S1. Average rate of exploration behaviours towards the surface (a) and the frame (b) of the apparatus, search behaviours (c) and looks down (d) towards the surface per minute exhibited by all three species in all conditions during the exposure phase.

**Supplement 3: Principal component analysis of the birds´ behaviours towards the mirror in the mirror exposure phase of the experiment**

1. *Data analysis*

We performed a principal component analysis in R (Version 4.0.0, R Core Team, 2020) using the package “FactorMineR” (Lê et al. 2008) including means of all behaviours performed by all 3 species in front of the mirror in the mirror exposure phase of the experiment. We excluded variables with no or close to no occurrences before starting the PCA and therefore retrained 10 variables. We split the dataset into 4 blocks à 3 mirrors sessions plus 1 category for the supplemental sessions per individual in order to reflect changes over time of exposure, therefore accounting for the behavioural changes linked to the increased experience with the mirror. The assumptions for the PCA were tested using the R package “ FactorAssumptions” (Stropoli 2020).

1. *Results of the PCA*

The assumptions for the PCA were met, Kaiser-Meyer-Olkin factor adequacy was of 0.56, Bartlett´s test of sparsity (Chi-squared= 251.56, df=45 p< 0.001; df=45) and in the communality check all variables had communality values above 0.70 (except for Time around that had an h2 score of 0.49). Based on the Eigenvalues (>1) and visual inspection of the scree-plot, we retained 5 factors, accounting for about 76% of the variance in the data (Table S1). Overall, dimension 1 seemed to be associated to the birds´ exploration behaviours (grouping mainly: Time in front, Time around, Peck Surface and Looking down), dimension 2 corresponds with contingency checking behaviours, dimension 3 with neophobic responses towards the mirror, dimension 4 with self-directed behaviours and dimension 5 with social behaviours (i.e. vocalizations). This general categorization largely supports the behavioural categorization used in this study and appears to be in line with the literature.

Table S2: Loadings of each variable included in the analysis for each retained component.

Salient loadings are highlighted in bold.

|  | **RC1** | **RC2** | **RC3** | **RC4** | **RC5** |
| --- | --- | --- | --- | --- | --- |
| Vocalization | 0.08 | -0.05 | -0.03 | -0.05 | **0.95** |
| Time in front | **0.40** | 0.26 | -0.02 | **0.52** | **0.48** |
| Time around | **0.67** | -0.05 | 0.12 | 0.15 | 0.06 |
| Peekaboo | -0.08 | **0.92** | -0.04 | 0.07 | 0.00 |
| Peck Surface | **0.82** | 0.13 | -0.07 | -0.05 | 0.07 |
| Peck frame | 0.17 | 0.00 | **0.81** | 0.09 | 0.10 |
| Look behind | 0.18 | **0.92** | 0.00 | 0.04 | -0.01 |
| Startle | 0.00 | -0.04 | **0.81** | -0.18 | -0.15 |
| Look down | **0.86** | 0.02 | 0.16 | -0.09 | 0.06 |
| Self-Directed Behaviour | -0.06 | 0.05 | -0.07 | 0.93 | -0.06 |
| Eigenvalues | 2.09 | 1.79 | 1.36 | 1.22 | 1.18 |
| Variance % | 0.21 | 0.18 | 0.14 | 0.12 | 0.12 |
| Cumulative % | 0.21 | 0.39 | 0.52 | 0.65 | 0.76 |
